# Cellugyrin (Synaptogyrin-2) Regulates Macrophage Phagocytosis of *Aggregatibacter actinomycetemcomitans* (*Aa*)

**DOI:** 10.3390/pathogens15050505

**Published:** 2026-05-08

**Authors:** Taewan J. Kim, Sherry Schneider, Aleena Defreitas, Lisa P. Walker, Bruce J. Shenker, Kathleen Boesze-Battaglia

**Affiliations:** 1 Department of Periodontics, School of Dental Medicine, University of Pennsylvania, Philadelphia, PA 19104, USA; taewank@upenn.edu; 2 Department of Basic and Translational Sciences, School of Dental Medicine, University of Pennsylvania, Philadelphia, PA 19104, USA; sherry31@upenn.edu (S.S.); aleenad@upenn.edu (A.D.); lism@upenn.edu (L.P.W.); shenker@upenn.edu (B.J.S.)

**Keywords:** *Aa*, C-MIP, cellugyrin, retrograde trafficking, Retro-2

## Abstract

Grade C molar-incisor pattern periodontitis (C-MIP) is a rapidly progressive form of periodontal disease affecting young individuals that is often linked to a highly virulent genotype of *Aggregatibacter actinomycetemcomitans* (*Aa*). Although *Aa* is present in the healthy oral microbiome, its transition into subgingival tissue correlates with the conversion from healthy to diseased status within the periodontal pocket. These changes may be due to immune evasion strategies attributed to *Aa* exotoxins. We previously demonstrated that a host cell protein, cellugyrin, plays a critical role in exotoxin internalization and subsequent cytotoxicity. Herein, we assess the contribution of cellugyrin to *Aa* phagocytosis and intracellular trafficking in human macrophages. Confocal imaging demonstrated that *Aa* co-localizes with cellugyrin. Importantly, cellugyrin-deficient macrophages exhibited a significant reduction in phagocytosed *Aa*. Furthermore, we analyzed the role of retrograde trafficking in *Aa* survival. Retro-2-mediated inhibition of this trafficking pathway resulted in increased intracellular *Aa*, likely due to increased survival. Collectively, our findings suggest that cellugyrin is involved in Aa phagocytosis and that retrograde trafficking may play a role in subsequent host cell clearance of *Aa*.

## 1. Introduction

Periodontal diseases encompass a complex spectrum of infectious and inflammatory conditions that compromise the gingiva, periodontal ligament, and alveolar bone, which together support the dentition [1]. Among these diseases, Grade C molar–incisor pattern periodontitis (C-MIP)—formerly termed localized aggressive periodontitis—stands out due to its rapid progression and severe tissue destruction at the molar–incisor sites [2]. Clinically, C-MIP is challenging to diagnose and manage because affected individuals often do not display overt signs of poor oral hygiene or generalized inflammation, yet frequently harbor *Aggregatibacter actinomycetemcomitans* (*Aa*), including highly leukotoxic JP2 strains [3]. These distinctive clinical features have prompted extensive research into the pathogenesis of C-MIP, with strong emphasis on microbial factors [4,5]. Among the implicated pathogens, *Aa* has emerged as a central contributor to aggressive forms of periodontitis [4,5].

Although *Aa* was initially regarded as the singular etiologic agent of C-MIP, it is now understood to operate within a broader dysbiotic microbial community [5]. *Aa* may exist as a benign oral commensal; however, when introduced into the anaerobic environment of the periodontal pocket, it transitions into a pathogenic phenotype [5]. Under these conditions, *Aa* employs multiple immune-evasion strategies, including the secretion of virulence factors such as the cytolethal distending toxin (Cdt) and leukotoxin A [5]. Cellugyrin (synaptogyrin-2), a member of the synaptogyrin tetraspanin protein family, is an essential host factor required for Cdt cytotoxicity [6,7]. Cellugyrin regulates the formation of synaptic-like microvesicles (SLMVs^Cg+^), which serve as intracellular transport vesicles [6,7,8,9]. These vesicles, associate with early sorting events at the trans-Golgi network, contain proteins involved in endocytic processing and lysosomal transport, including GLUT4 and phosphatidylinositol 4-kinase IIα [10,11,12]. Several adaptive variants of cellugyrin have identified this protein as a viral-interacting protein linking cellugyrin-associated pathways to viral infection [13]. Porcine circovirus 2 (PCV2)-associated disease variation in incidence and severity has been linked to missense mutations in the SYNGR2 gene (cellugyrin), resulting in viremia [14]. Sun et al., also reported on the role of cellugyrin in the infection of mammalian cells by Bunyavirus [15]. Notably, we have reported that infection of Calu-3 cells by SARS-CoV-2 and vesicular stomatitis virus (VSV) involves hijacking of the cellugyrin-dependent pathways to access intracellular compartments [16].

The role of cellugyrin in bacterial infection is unknown. These studies begin to fill this void. Herein, we sought to determine if phagocytosis and the subsequent killing of *Aa* in macrophages are dependent upon cellugyrin. First, we examined the requirement for cellugyrin in mediating *Aa* phagocytosis. Second, we utilized Retro-2 to determine if endosome-to-Golgi retrograde transport played a role in bacterial survival. Together, these investigations provide new insights into how *Aa* may hijack host vesicular systems and highlight potential therapeutic targets for managing aggressive forms of periodontitis.

## 2. Materials and Methods

### 2.1. Cell Culture

The THP-1 human acute monocytic leukemia cell line (TIB-202, ATCC; Manassas, VA, USA) was cultured in RPMI 1640 medium supplemented with 10% FBS, 1 mM sodium pyruvate, 20 µM 2-mercaptoethanol, and 2% penicillin-streptomycin at 37 °C with 5% CO_2_ in a humidified incubator. THP-1 monocytes with stable shRNA expression targeting cellugyrin and control cells were generated using lentiviral particles from Santa Cruz Biotech (Dallas, TX, USA) as we have described previously [7]. The transduction of THP-1 cells with these particles and non-target controls followed established protocols, with expression levels verified by Western blotting [6,7]. THP-1 monocytes were differentiated into macrophages (TDM) by treating with 50 ng/mL phorbol 12-myristate 13-acetate (PMA) for 48 h, followed by washing and incubation in fresh medium for an additional 24 h before use. Cells were routinely used prior to passage number 15 and screened for mycoplasma, with MycoAlert (Cambrex, East Rutherford, NJ, USA) by the Cell Center Service Facility at the University of Pennsylvania, monthly or as needed.

### 2.2. Bacteria and Growth Curve

*A. actinomycetemcomitans* (*Aa*) strain D7S-SA (wild-type *Aa*) was grown as described by Nalbant, A. et al. and in our previous studies and provided by Casey Chen at the University of Southern California [17,18,19]. *Aa* was plated on AAGM agar which consisted of 20 g of BBL trypticase soy agar (Becton Dickinson; Sparks, MD, USA) and 3 g of yeast extract (ThermoFisher; Waltham, MA, USA) supplemented with 0.4% sodium bicarbonate and 0.8% dextrose [18,19]. As previously described, after bacteria were grown on plates for 48 h in the incubator with 10% CO_2_ at 37 °C, they were then inoculated in 10 mL of AAGM broth until OD_600_ was close to 0.2 Absorbance Unit [18,19]. Bacteria were plated from different dilutions at various time points on AAGM agar plates and incubated for 24 h in the incubator with 10% CO_2_ at 37 °C [18,19]. OD_600_ was measured using a DU 650 Spectrophotometer (Beckman Coulter, Indianapolis, IN, USA); for each time point we concurrently determined the number of colony-forming units (CFUs) to determine the growth curve.

### 2.3. Bacteria Inoculation

TDMs (1 × 10^6^ cells per well) were differentiated in 12-well plates (ThermoFisher; Waltham, MA, USA) or 35 mm glass-bottom dishes (MatTek; Ashland, MA, USA) and then incubated with medium alone or with 100 uM Retro-2 (Sigma Aldrich; Burlington, MA, USA) for 30 min before bacterial inoculation. Before inoculation, the macrophages were washed three times with cell media lacking penicillin/streptomycin. As previously described, *Aa* was grown to the logarithmic phase (OD_600_ between 0.5 and 1 Absorbance Unit) at 37 °C with 10% CO_2_ and used to inoculate the macrophages at a multiplicity of Infection (MOI) of 1:100 (TDM to bacteria) for imaging and 1:10 for survival study (for 2, 4, 6 and 8 h) at 37 °C with 5% CO_2_ in penicillin/streptomycin-free media with and without 100 µM Retro-2 [7,18,19]. After incubation, cells were washed three times with PBS and treated with gentamicin (50 μg/mL) to eliminate extracellular bacteria [18,19]. Soy broth containing 1% saponin (200 μL per well) was added to lyse macrophages [18,19]. The lysates were plated (100 μL per plate) on AAGM agar plates and incubated for 2 days at 37 °C with 10% CO_2_. The number of surviving bacteria was determined by counting CFUs [18,19].

### 2.4. Western Blot Analysis

Macrophages were treated as described above and solubilized in 20 mM Tris-HCl buffer (pH 7.5) containing 150 mM NaCl, 1 mM EDTA, 1% NP-40, 1% sodium deoxycholate and protease inhibitor cocktail (ThermoFisher Scientific; Waltham, MA, USA). Samples (30 µg) were separated on 4–12% SDS-PAGE and then transferred to PVDF membranes. The membranes were blocked with BLOTTO and then incubated with anti-cellugyrin (1:1000, prepared as previously reported [6]) primary antibodies overnight at 4 °C as previously described [18,19]. Membranes were washed and incubated with species-matching secondary immunoglobulin conjugated to horseradish peroxidase (Southern Biotech Technology; Birmingham, AL, USA). The Western blots were developed using chemiluminescence and analyzed by digital densitometry (Li Cor Biosciences; Lincoln, NE, USA) as previously described [18,19].

### 2.5. Confocal Microscopy

Analysis of cellugyrin association with *Aa* was completed as follows. TDMs were inoculated with *Aa* for 1 h as described above. After inoculation, cells were washed three times with PBS and fixed in cold methanol (−20 °C, 15 min) for cellugyrin staining, washed again, and processed for immunostaining with anti-cellugyrin (1:100, prepared as previously reported [6]). Briefly, the cells were blocked in blocking solution containing 5% BSA and 0.2% Triton X-100 in PBS (PBST) at 37 °C for 1 h and incubated with anti-cellugyrin antibody diluted in blocking solution at 4 °C overnight [7]. The next day, they were washed three times with PBST and incubated with appropriate secondary antibodies (1:1000) and Hoechst 33258 (1:5000, Invitrogen, Waltham, MA, USA) diluted in blocking solution for 2 h and at room temperature. Samples were ready for imaging after three washes with PBS [7]. For *Aa* phagocytosis studies, the inoculation protocol described above was followed. Cells were washed three times with PBS and fixed in 4% paraformaldehyde and washed again. *Aa* staining was done with anti-*Aa* (1:100, 150AA1.1 deposited to the Developmental Studies Hybridoma Bank by Gmuer, R) and the rest of the procedure was the same as for cellugyrin. For all the confocal fluorescence staining, secondary-only controls were done to confirm non-specific background and actual signals.

**Figure 2:**
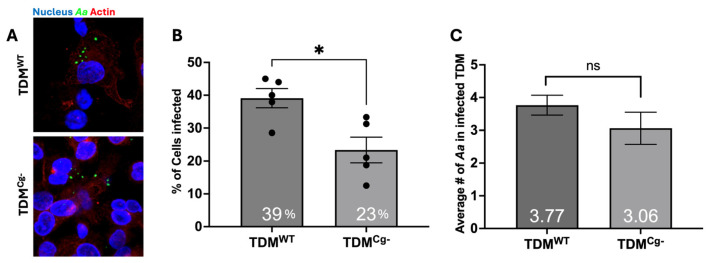
**Cellugyrin’s role in *Aa* phagocytosis.** TDM^WT^ and TDM^Cg−^ were inoculated with *Aa* with MOI of 1:100 for 1 h and treated as described in the Methods and percent of TDM infected with *Aa* and average number of *Aa* per TDM was analyzed by confocal images. (**A**) Representation images of *Aa*-infected TDM^WT^ and TDM^Cg−^, nucleus (blue), *Aa* (green) stained by *Aa* antibody (150AA1.1), and actin (red). (**B**) Percentage of TDMs containing *Aa.* (**C**) Average number of *Aa* per *Aa*-positive TDMs. In (**B**,**C**), mean ± standard error of the mean of blinded counts within 0.13 mm by 0.13 mm regions from 3 biological replicate data were analyzed using Student’s *t*-test (*, *p* < 0.05), ns—not significant.

Images were captured on a Nikon A1R laser scanning confocal microscope equipped with a PLAN APO VC 100×oil (NA 1.45) objective at room temperature. Z-stacks were acquired at an interval of 0.1 μm (50 focal planes/image stack, 5 μm), and data were analyzed using Nikon NIS-Elements AR version 4.30.01 software. Standard LUT adjustments were used for the image presentation. For quantification of *Aa*–cellugyrin association, fluorescence intensity of cellugyrin staining surrounding *Aa*, detected as Hoechst-positive structures less than 2 μm in diameter, was measured. DNA-rich structures, including *Aa* and TDM nuclei, were stained with Hoechst 33258 (Invitrogen, Waltham, MA, USA) [20]. *Aa* internalization was confirmed using the anti-*Aa* antibody described above and is shown in Figure 2A. The *Aa* antibody could not be used concurrently with the anti-cellugyrin antibody due to differences in fixation requirements. All the experiments were done in 3 biological replicates, and 5 *Aa* that were phagocytosed by TDMs were randomly selected. A 4 μm^2^ area (2 μm × 2 μm) surrounding *Aa* was defined as the region of interest (ROI), and the summed fluorescence intensity of cellugyrin across the z-stack was used for quantification, as described in our previous work [18]. For quantification of *Aa* and *Aa*-infected TDMs, five random fields were selected from either the top-center or bottom-center regions, to ensure no edge effects, across three independent biological replicates. Within each field, TDMs containing phagocytosed *Aa* were identified, and intracellular *Aa* were quantified by direct counting and analyses were performed blindly.

### 2.6. Statistical Analysis

All statistical analyses were performed using GraphPad Prism version 10.0.3 (GraphPad, Boston, MA, USA) with an alpha value of 0.05. Means were compared using a *t*-test or two-way ANOVA with Tukey’s multiple comparisons post hoc test as indicated in figure legends. All data were graphed as mean ± standard error of the mean (SEM), unless otherwise indicated. *p*-values were represented as: * *p* < 0.05, ** *p* < 0.01, *** *p* < 0.001, ns *p* > 0.05.

## 3. Results

### 3.1. Involvement of Cellugyrin in Aa Phagocytosis by Human Macrophages

**Figure 1:**
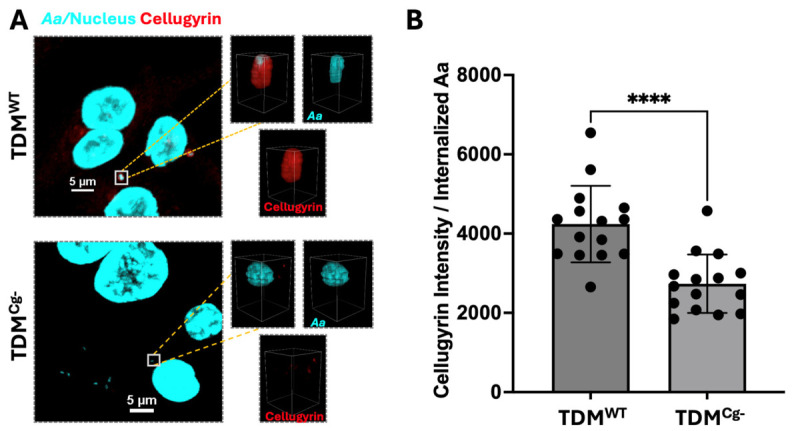
***Aa* co-distributes with Cellugyrin.** TDM^WT^ and TDM^Cg−^ were inoculated with *Aa* (MOI of 1:100) for 1 h and treated as described in the Methods. (**A**) Representative multi-fluor confocal images showing *Aa* (pseudocolored-cyan), cellugyrin (red), and nuclei (pseudocolored-cyan) with 2 μm by 2 μm volumetric reconstruction around *Aa*. Data in (**B**) represent raw cellugyrin fluorescence intensity around the *Aa*. Data in (**B**) are presented as mean ± standard error of the mean (5 *Aa* per each of three biological replicates were analyzed, for n = 15) and analyzed using Student’s *t*-test (****, *p* < 0.0001).

We previously established that cellugyrin-deficient macrophages exhibited reduced Cdt internalization and were resistant to Cdt-induced toxicity [7,8]. We now extend these studies to determine if phagocytosed *Aa* associates with cellugyrin. THP-1-derived macrophages (TDM), both wild-type (TDM^WT^) and cellugyrin-deficient TDM (TDM^Cg−^), were challenged with *Aa* at a multiplicity of infection (MOI) of 1:100 (TDM:*Aa*) for 1 h. We first determined if phagocytosed *Aa* co-localized with cellugyrin. As shown in Figure 1A, *Aa* was found to be associated with cellugyrin-positive structures as illustrated by 3D reconstruction using multi-fluor confocal imaging. Semi-quantitative assessment of cellugyrin fluorescence revealed a strong signal surrounding *Aa* in TDM^WT^, suggesting that cellugyrin is recruited to *Aa*-containing phagosomes and co-localizes with the bacterium during phagocytosis, suggesting a functional interaction in host–pathogen interactions. *Aa* did not alter the levels of cellugyrin expression (Figure S1).

Next, we determined if cellugyrin was required for *Aa* phagocytosis. Both wild-type (TDM^WT^) and cellugyrin-deficient TDM (TDM^Cg−^) were challenged with *Aa* at an MOI of 1:100 for 1 h. The percent of TDMs containing phagocytosed *Aa* and the number of *Aa* per TDM were determined (Figure 2). The TDM^WT^ population exhibited 39 ± 2.9% *Aa* positive cells; this compared to 23 ± 3.9% in the TDM^Cg−^ population (Figure 2B), suggesting that cellugyrin contributes to phagocytic efficiency. The number of *Aa* cells phagocytosed per *Aa*-positive TDM was also determined (Figure 2C). The average number of phagocytosed *Aa* per *Aa*-positive TDMs was 3.77 ± 0.3 in TDM^WT^; impaired expression of cellugyrin slightly reduced the number of phagocytosed bacteria to 3.06 ± 0.49; this reduction was not observed to be statistically significant.

**Figure 3:**
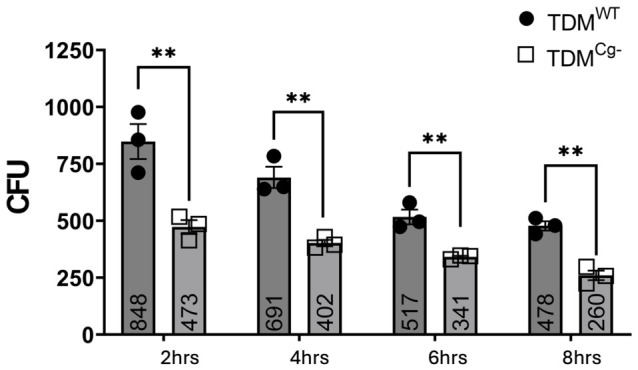
**Effect of impaired cellugyrin expression on phagocytosis.** TDM^WT^ and TDM^Cg−^ were inoculated with *Aa* for 2, 4, 6, and 8 h. Cell lysates were plated on agar plate and CFUs were determined as described in Methods. Data represent (bar graph and numeric value) mean +/− standard error (n = 3, biological replicates) CFU at each time points (2, 4, 6, and 8 h) and were compared using two-way ANOVA (**, *p* < 0.01).

In the next series of experiments, we determined whether cellugyrin was required for the bactericidal capacity of macrophages. TDMs were inoculated with *Aa* at a multiplicity of infection, MOI 1:10, for 2, 4, 6, and 8 h; the vitality of the phagocytosed *Aa* was subsequently determined by measuring colony-forming units (CFUs). TDM^WT^ macrophages exhibited 848 ± 77 CFU *Aa* survival at 2 h, which decreased to 691 ± 47 CFU at 4 h, 517 ± 32 CFU at 6 h, and 478 ± 20 CFU by 8 h. In contrast, TDM^Cg−^ macrophages showed 473 ± 38 CFU at 2 h, dropping to 402 ± 14 CFU at 4 h, 341 ± 5 CFU at 6 h, and 260 ± 21 CFU at 8 h (Figure 3). At all time points, *Aa* survival was significantly lower (*p*-value < 0.05 2 h) in TDM^Cg−^ cells compared to TDM^WT^. When we compared the percent total decrease in CFU upon *Aa* treatment, we found that the overall decrease in *Aa* was similar between the two groups: TDM^WT^ had about a 56% decrease and TDM^Cg−^ had about a 55% decrease by 8 h. These results suggest that cellugyrin plays a role in phagocytosis, but less so in *Aa* trafficking and subsequent bacterial killing.

### 3.2. Retrograde Transport and Bacterial Survival in Macrophages

**Figure 4:**
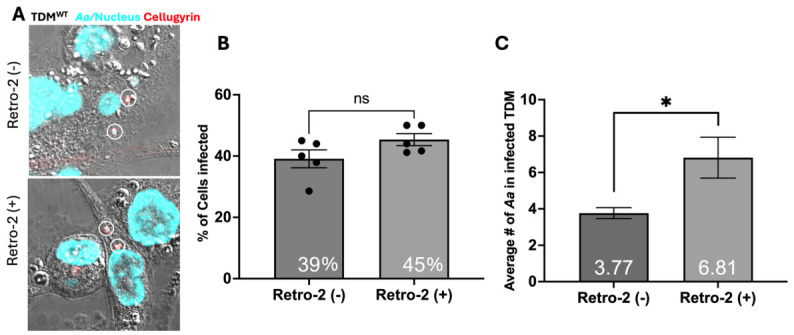
**Retro-2 treatment increases *Aa* in TDM^WT^.** TDM^WT^ were pre-treated with Retro-2 100 µM (Retro-2 (+)) for 30 min or left-untreated, control (Retro-2 (−), same data as Figure 2). Cells were subsequently inoculated with *Aa* for 1 h. (**A**) Representative multi-fluor confocal images. For images, cell nuclei and *Aa* stained with DAPI (pseudocolored-cyan) and cellugyrin (red) as in Methods. Five random images from 3 independent experiments were used to analyze: (**B**) Percentage of TDM^WT^ cells containing *Aa* and (**C**) Average number of *Aa* per *Aa*-positive TDM^WT^. Data represent (bar graph and numeric value) mean ± standard error of the mean of blinded counts within 0.13 mm by 0.13 mm regions from 3 biological replicates and were compared using Student’s *t*-test (*, *p*< 0.05), ns—not significant.

**Figure 5:**
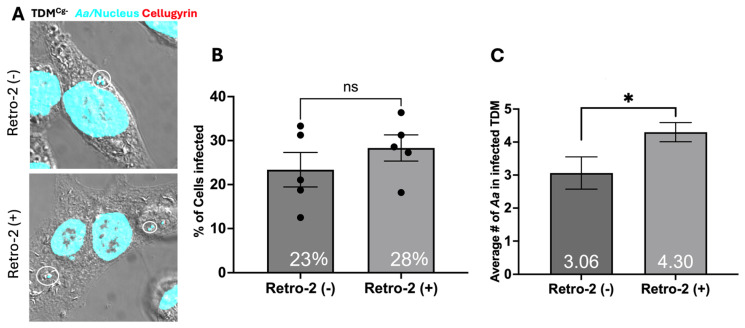
**Retro-2 treatment increases *Aa* in TDM^Cg−^.** TDM^Cg−^ was pre-treated with Retro-2 100 µM (Retro2 (+)) for 30 min or left untreated, control (Retro-2 (−), same data as Figure 2). Cells were subsequently inoculated with *Aa* for 1 h. (**A**) Representative multi-fluor confocal images, cell nuclei and *Aa* stained with DAPI (pseudocolored-cyan) and cellugyrin (red) as in Methods. Five random images from 3 independent experiments were used to analyze (**B**) Percentage of TDM^Cg−^ cells containing *Aa* and (**C**) Average number of *Aa* per *Aa*-positive TDM^Cg−^. Data represent (bar graph and numeric value) mean ± standard error of the mean of blinded counts within 0.13 mm by 0.13 mm regions from 3 biological replicates and were compared using Student’s *t*-test (*, *p* < 0.05), ns—not significant.

To assess the requirement for cellugyrin in retrograde trafficking and subsequent bactericidal activity, we utilized Retro-2 to inhibit retrograde transport. Retro-2 inhibits endosome-to-Golgi retrograde transport, blocking the trafficking of toxins such as Cdt, ricin, and Shiga toxin, and inhibits the invasion of intracellular parasites, non-enveloped viruses, and autolysosome formation [16,21,22]. Using multi-fluor confocal imaging, we assessed the intracellular distribution of *Aa* in the presence or absence of Retro-2 (Figure 4A and Figure 5A). Pre-treatment with Retro-2 had no effect on the percentage of *Aa* phagocytosed by TDM^WT^ (percent of *Aa*-containing TDM in five random fields: untreated 39 ± 2.9% and treated 45 ± 2.0%) or TDM^Cg−^ (untreated 23 ± 2.9% and treated 28 ± 3.9%) (Figure 4B and Figure 5B). Retro-2-treatment resulted in a significant increase in the average number of *Aa* per cell. TDM^WT^ treated with Retro-2 cells showed almost double the number of *Aa* per cell, with 6.8 ± 1.12 *Aa* per cell compared to 3.8 ± 0.3 *Aa* per cell in untreated TDM^WT^ (Figure 4C). TDM^Cg−^ also showed a significant increase of 4.3 ± 0.29 *Aa* per cell with Retro-2 treatment versus 3.0 ± 0.49 *Aa* per cell without treatment (Figure 5C). The increase in the number of *Aa* per TDM is consistent with a requirement for endosomal transport for macrophage-mediated bacterial survival.

**Figure 6:**
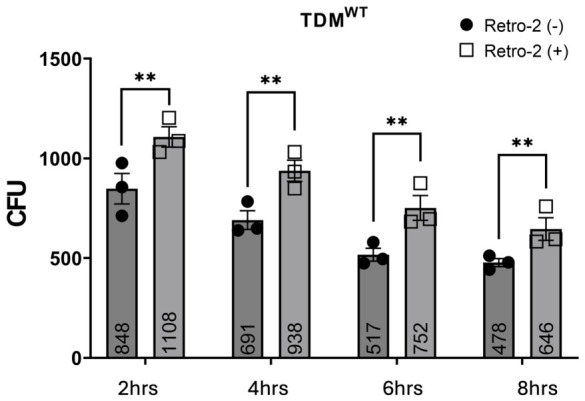
**Retro-2 modulates bactericidal capacity of Macrophages.** TDM^WT^ pre-treated with Retro-2 for 30 min or no treatment (Retro-2 (−)) were inoculated with *Aa* for 2, 4, 6, and 8 h. Cell lysates were plated on agar plate and CFU were determined as described in Methods. Data (bar graph and numeric value) represent mean and +/− standard error (n = 3, biological replicates) CFU at each time points (2, 4, 6, and 8 h) and compared using two-way ANOVA (**, *p* < 0.01).

To assess the role of retrograde transport in *Aa* survival, TDM^WT^ and TDM^Cg−^ macrophages were either pre-treated with Retro-2 for 30 min or left untreated, followed by inoculation with *Aa* (MOI 1:10) for 2, 4, 6, and 8 h. In untreated TDM^WT^ cells, *Aa* survival was 848 ± 77 CFU at 2 h, decreasing to 691 ± 47 CFU at 4 h, 517 ± 32 CFU at 6 h, and 478 ± 20 CFU by 8 h. In contrast, Retro-2-treated TDM^WT^ cells exhibited significantly higher *Aa* survival: 1108 ± 51 CFU at 2 h, decreasing to 938 ± 53 CFU at 4 h, 752 ± 62 CFU at 6 h, and 646 ± 57 CFU at 8 h (Figure 6).

**Figure 7:**
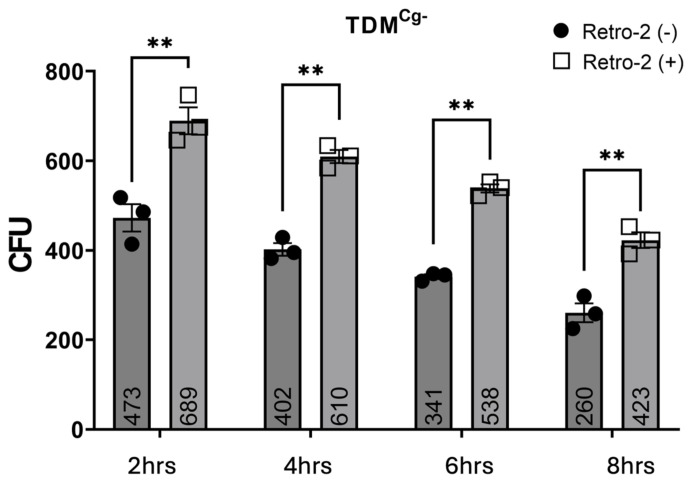
**Retro-2 modulates bactericidal capacity of cellugyrin-silent Macrophages.** TDM^Cg−^ pre-treated with Retro-2 for 30 min or no treatment (Retro-2 (−)) were inoculated with *Aa* for 2, 4, 6, and 8 h. Cell lysates were plated on agar plate and CFU were determined as described in Methods. Data (bar graph and numeric value) represent mean +/− standard error (n = 3, biological replicates) CFU at each time points (2, 4, 6, and 8 h) and compared using two-way ANOVA (**, *p* < 0.01).

In untreated TDM^Cg−^ cells, Aa survival was 473 ± 31 CFU at 2 h, decreasing to 402 ± 14 CFU at 4 h, 341 ± 5 CFU at 6 h, and 260 ± 21 CFU by 8 h. In contrast, Retro-2-treated TDM^Cg−^ cells exhibited significantly higher Aa survival: 689 ± 30 CFU at 2 h, decreasing to 610 ± 14 CFU at 4 h, 538 ± 9 CFU at 6 h, and 423 ± 18 CFU at 8 h (Figure 7). Across all time points, Retro-2 treatment resulted in significantly increased Aa CFUs in both TDM^WT^ and TDM^Cg−^ cells compared to untreated controls. However, despite the higher bacterial burden, the temporal pattern of decline remained comparable between groups.

## 4. Discussion

**Figure 8:**
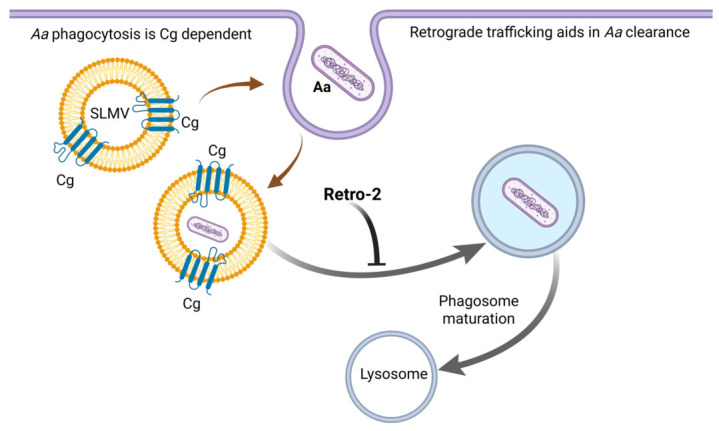
**Schematic representation of host–cell pathways utilized by *Aggregatibacter actinomycetemcomitans (Aa)* during uptake and trafficking**. Our studies suggest that cellugyrin (Cg)-positive vesicles termed synaptic-like microvesicles (SLMV^Cg^) may facilitate *Aa* uptake. SLMV^Cg^ are recruited to plasma membrane as indicated by brown arrows. Subsequent trafficking appears to utilize retrograde transport, inhibition of this transport mechanism by Retro-2 contributes to *Aa* survival.

This study provides new insights into the molecular mechanisms by which *Aa* interacts with host macrophages, highlighting the roles of cellugyrin and endosome-to-Golgi retrograde trafficking in *Aa* phagocytosis and survival. Building upon prior research demonstrating the involvement of cellugyrin in Cdt internalization and function, we investigated whether *Aa* similarly exploits cellugyrin-mediated pathways during phagocytosis [6,7,8]. Our findings show that cellugyrin not only co-localizes with intracellular *Aa* but that its presence significantly enhances *Aa* uptake by macrophages as well. Cellugyrin-deficient macrophages (TDM^Cg−^) exhibited a marked decrease in the number of cells containing phagocytosed Aa and a reduced number of intracellular bacteria per cell compared to wild-type (TDM^WT^) controls (Figure 2A). These results support a model in which cellugyrin-associated vesicular structures may facilitate early stages of *Aa* internalization or trafficking within the host cell (Figure 8).

Interestingly, while cellugyrin appears to be utilized for *Aa* phagocytosis, it does not appear to be necessary for the bactericidal function of macrophages. In this regard, CFU analysis showed that TDM^Cg−^ macrophages had significantly lower intracellular *Aa* survival at all time points compared to TDM^WT^ cells, despite exhibiting similar clearance kinetics. This suggests that although cellugyrin facilitates *Aa* entry, it does not contribute to the formation of intracellular compartments that are less degradative or protective, which would otherwise allow *Aa* to evade lysosomal fusion and persist within host cells. These findings are consistent with our prior reports showing that *Aa* can manipulate host vesicular trafficking to evade immune responses [18], but only with Cdt internalization, which requires cellugyrin [6,7,8].

To further explore whether *Aa* hijacks retrograde trafficking pathways for intracellular persistence, we used Retro-2, a small-molecule inhibitor of endosome-to-Golgi transport. While Retro-2 treatment did not significantly alter the proportion of macrophages containing phagocytosed *Aa*, it led to a substantial increase in the average number of *Aa* per cell in both TDM^WT^ and TDM^Cg−^. Moreover, CFU counts revealed that Retro-2-treated TDM^WT^ cells exhibited significantly higher intracellular *Aa* survival at all time points compared to untreated controls. These data indicate that inhibition of retrograde trafficking impairs the bactericidal capacity of macrophages, allowing for increased intracellular retention of *Aa* as illustrated in Figure 8.

The observed increase in bacterial burden following Retro-2 treatment suggests that retrograde trafficking may play a functional role in the maturation of phagosomes and in bacterial degradation. This is consistent with prior findings with other intracellular pathogens and toxins, where Retro-2 disrupts trafficking to the Golgi and blocks downstream processing in endolysosomal compartments [7,21,22]. In the context of *Aa* infection, our findings support the hypothesis currently under investigation that endosome-to-Golgi trafficking modulates phagosome-lysosome fusion or delivery of antimicrobial effectors to the phagosome. Interference with this pathway compromises bacterial clearance and promotes *Aa* survival.

Taken together, our data demonstrates that both cellugyrin and retrograde trafficking pathways contribute to *Aa*-host interactions. Cellugyrin appears to facilitate *Aa* uptake, possibly through the formation of specialized vesicles that support internalization, while intact retrograde transport is essential for the subsequent bactericidal response. These findings contribute to a growing body of literature describing how *Aa* manipulates host cell machinery for immune evasion and survival [18,23]. They also suggest that targeting these trafficking pathways may represent a novel strategy to enhance innate immune clearance of *Aa*, offering potential therapeutic implications for C-MIP.

While our findings shed light on important aspects of *Aa* uptake and intracellular processing, this study is limited by its reliance on in vitro macrophage models, which do not fully capture the complexity of periodontal tissues. Future in vivo studies and evaluation of additional host cell types will be essential to determine the broader relevance of these pathways. Nevertheless, these results provide a strong foundation for exploring therapeutic strategies that target cellugyrin- or retrograde-trafficking pathways to enhance host clearance of *Aa* and potentially improve outcomes in C-MIP.

## Data Availability

The original contributions presented in the study are included in the article and presented as either primary or supplemental data. The data that support the findings of this study including raw image files, numerical datasets, and uncropped blot files are available from the corresponding author upon reasonable request.

## Supplementary Materials

The following supporting information can be downloaded at https://www.mdpi.com/article/10.3390/pathogens15050505/s1, Figure S1. *Aa* does not alter levels of intracellular cellugyrin.

## Abbreviations

The following abbreviations are used in this manuscript:

C-MIP	Grade C molar-incisor pattern periodontitis
*Aa*	*Aggregatibacter actinomycetemcomitans*
Cdt	cytolethal distending toxin
SLMVs^Cg+^	synaptic-like microvesicles
TDM	THP-1 monocytes derived macrophages
MOI	Multiplicity of Infection
CFU	colony-forming units

## References

[1] Teughels W., Dhondt R., Dekeyser C., Quirynen M. (2014). Treatment of aggressive periodontitis. Periodontology 2000.

[2] Tonetti M.S., Greenwell H., Kornman K.S. (2018). Staging and grading of periodontitis: Framework and proposal of a new classification and case definition. J. Periodontol..

[3] Kachlany S.C., Fine D.H., Figurski D.H. (2000). Secretion of RTX leukotoxin by Actinobacillus actinomycetemcomitans. Infect. Immun..

[4] Fine D.H., Markowitz K., Furgang D., Velliyagounder K. (2010). Aggregatibacter actinomycetemcomitans as an early colonizer of oral tissues: Epithelium as a reservoir?. J. Clin. Microbiol..

[5] Fine D.H., Patil A.G., Velusamy S.K. (2019). Aggregatibacter actinomycetemcomitans (Aa) Under the Radar: Myths and Misunderstandings of Aa and Its Role in Aggressive Periodontitis. Front. Immunol..

[6] Boesze-Battaglia K., Walker L.P., Dhingra A., Kandror K., Tang H.Y., Shenker B.J. (2017). Internalization of the Active Subunit of the Aggregatibacter actinomycetemcomitans Cytolethal Distending Toxin Is Dependent upon Cellugyrin (Synaptogyrin 2), a Host Cell Non-Neuronal Paralog of the Synaptic Vesicle Protein, Synaptogyrin 1. Front. Cell. Infect. Microbiol..

[7] Boesze-Battaglia K., Dhingra A., Walker L.M., Zekavat A., Shenker B.J. (2020). Internalization and Intoxication of Human Macrophages by the Active Subunit of the Aggregatibacter actinomycetemcomitans Cytolethal Distending Toxin Is Dependent Upon Cellugyrin (Synaptogyrin-2). Front. Immunol..

[8] Shenker B.J., Korostoff J., Walker L.P., Zekavat A., Dhingra A., Kim T.J., Boesze-Battaglia K. (2024). Aggregatibacter actinomycetemcomitans Cytolethal Distending Toxin Induces Cellugyrin-(Synaptogyrin 2) Dependent Cellular Senescence in Oral Keratinocytes. Pathogens.

[9] Belfort G.M., Bakirtzi K., Kandror K.V. (2005). Cellugyrin induces biogenesis of synaptic-like microvesicles in PC12 cells. J. Biol. Chem..

[10] Kupriyanova T.A., Kandror K.V. (2000). Cellugyrin is a marker for a distinct population of intracellular Glut4-containing vesicles. J. Biol. Chem..

[11] Kioumourtzoglou D., Pryor P.R., Gould G.W., Bryant N.J. (2015). Alternative routes to the cell surface underpin insulin-regulated membrane trafficking of GLUT4. J. Cell Sci..

[12] Xu Z., Huang G., Kandror K.V. (2006). Phosphatidylinositol 4-kinase type IIalpha is targeted specifically to cellugyrin-positive glucose transporter 4 vesicles. Mol. Endocrinol..

[13] Walker L.R., Vu H.L., Montooth K.L., Ciobanu D.C. (2023). Functional and evolutionary analysis of host Synaptogyrin-2 in porcine circovirus type 2 susceptibility. PLoS Genet..

[14] Walker L.R., Engle T.B., Vu H., Tosky E.R., Nonneman D.J., Smith T.P.L., Borza T., Burkey T.E., Plastow G.S., Kachman S.D. (2018). Synaptogyrin-2 influences replication of Porcine circovirus 2. PLoS Genet..

[15] Sun Q., Qi X., Zhang Y., Wu X., Liang M., Li C., Li D., Cardona C.J., Xing Z. (2016). Synaptogyrin-2 Promotes Replication of a Novel Tick-borne Bunyavirus through Interacting with Viral Nonstructural Protein NSs. J. Biol. Chem..

[16] Boesze-Battaglia K., Cohen G.H., Bates P.F., Walker L.M., Zekavat A., Shenker B.J. (2024). Cellugyrin (synaptogyrin-2) dependent pathways are used by bacterial cytolethal distending toxin and SARS-CoV-2 virus to gain cell entry. Front. Cell. Infect. Microbiol..

[17] Nalbant A., Chen C., Wang Y., Zadeh H.H. (2003). Induction of T-cell apoptosis by Actinobacillus actinomycetemcomitans mutants with deletion of ltxA and cdtABC genes: Possible activity of GroEL-like molecule. Oral Microbiol. Immunol..

[18] Kim T.J., Shenker B.J., MacElory A.S., Spradlin S., Walker L.P., Boesze-Battaglia K. (2023). Aggregatibacter actinomycetemcomitans cytolethal distending toxin modulates host phagocytic function. Front. Cell. Infect. Microbiol..

[19] Kim T.J., MacElroy A.S., Defreitas A., Shenker B.J., Boesze-Battaglia K. (2024). A Synthetic Small Molecule, LGM2605: A Promising Modulator of Increased Pro-Inflammatory Cytokine and Osteoclast Differentiation by Aggregatibacter actinomycetemcomitans Cytolethal Distending Toxin. Dent. J..

[20] Vicencio E., Cordero E.M., Cortes B.I., Palominos S., Parra P., Mella T., Henrriquez C., Salazar N., Monasterio G., Cafferata E.A. (2020). Aggregatibacter Actinomycetemcomitans Induces Autophagy in Human Junctional Epithelium Keratinocytes. Cells.

[21] Nicolas V., Lievin-Le Moal V. (2020). Small Trafficking Inhibitor Retro-2 Disrupts the Microtubule-Dependent Trafficking of Autophagic Vacuoles. Front. Cell Dev. Biol..

[22] Gupta N., Noel R., Goudet A., Hinsinger K., Michau A., Pons V., Abdelkafi H., Secher T., Shima A., Shtanko O. (2017). Inhibitors of retrograde trafficking active against ricin and Shiga toxins also protect cells from several viruses, Leishmania and Chlamydiales. Chem. Biol. Interact..

[23] Ando-Suguimoto E.S., da Silva M.P., Kawamoto D., Chen C., DiRienzo J.M., Mayer M.P. (2014). The cytolethal distending toxin of Aggregatibacter actinomycetemcomitans inhibits macrophage phagocytosis and subverts cytokine production. Cytokine.

