# Supplementary figures and images for: Cellugyrin (Synaptogyrin-2) Regulates Macrophage Phagocytosis of Aggregatibacter actinomycetemcomitans (Aa)
Source: Pathogens. 2026 May 8;15(5):505. doi: 10.3390/pathogens15050505 (PMC13210048; doi:10.3390/pathogens15050505)

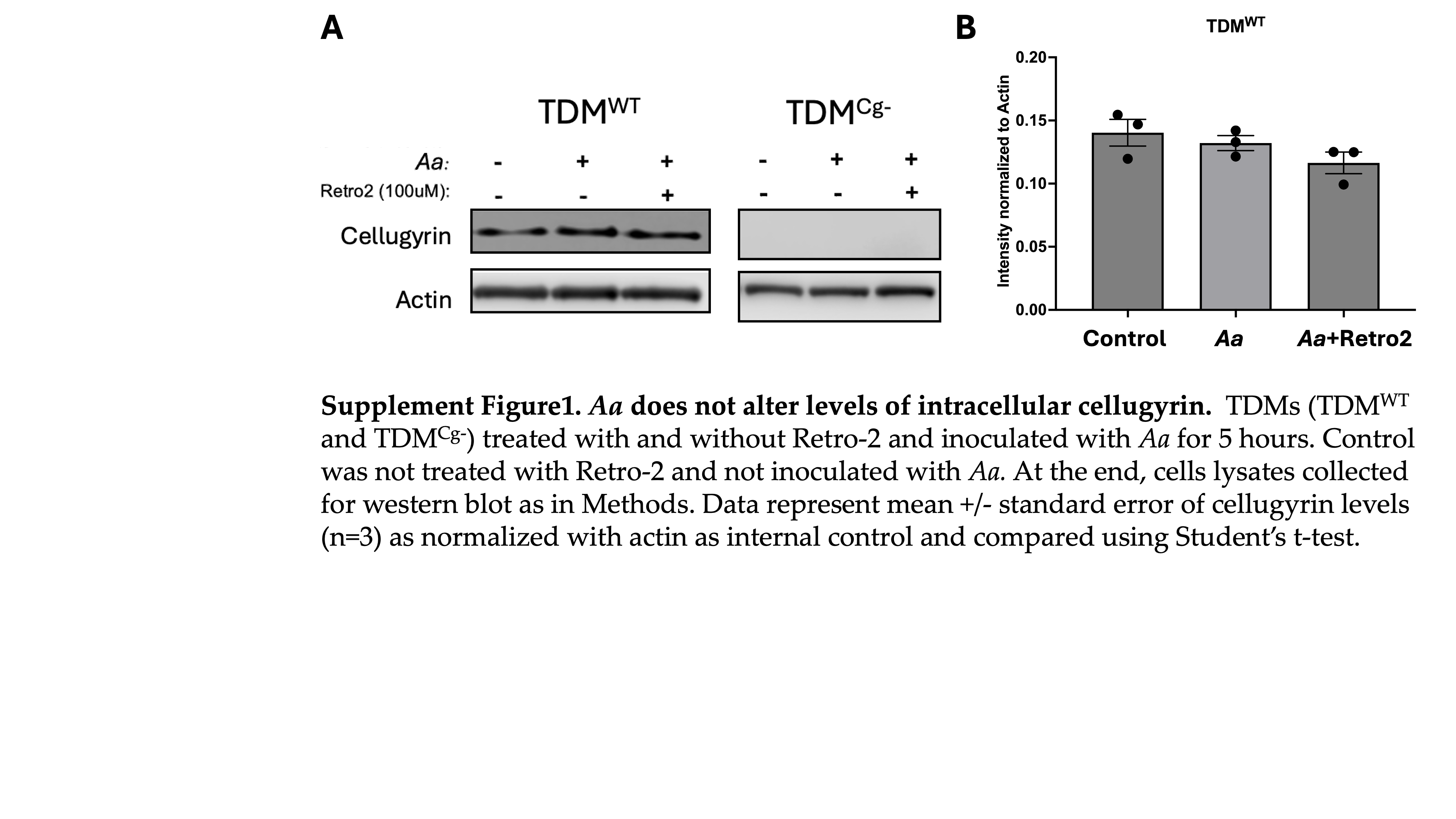

Supplement: Supplementary file 1 [file pathogens-15-00505-s001.zip › pathogens-4270972-supplementary.tiff]
